# Coherent activity within and between hemispheres: cortico-cortical connectivity revealed by rTMS of the right posterior parietal cortex

**DOI:** 10.3389/fnhum.2024.1362742

**Published:** 2024-03-07

**Authors:** Chiara Mazzi, Sonia Mele, Chiara Bagattini, Javier Sanchez-Lopez, Silvia Savazzi

**Affiliations:** ^1^Perception and Awareness (PandA) Laboratory, Department of Neuroscience, Biomedicine and Movement Sciences, University of Verona, Verona, Italy; ^2^Section of Neurosurgery, Department of Neuroscience, Biomedicine and Movement Sciences, University of Verona, Verona, Italy; ^3^Escuela Nacional de Estudios Superiores Unidad Juriquilla, Universidad Nacional Autonoma de Mexico, Santiago de Querétaro, Mexico

**Keywords:** resting state, EEG oscillatory dynamics, functional connectivity, inter-hemispheric coherence, rTMS

## Abstract

**Introduction:**

Low frequency (1 Hz) repetitive transcranial stimulation (rTMS) applied over right posterior parietal cortex (rPPC) has been shown to reduce cortical excitability both of the stimulated area and of the interconnected contralateral homologous areas. In the present study, we investigated the whole pattern of intra- and inter-hemispheric cortico-cortical connectivity changes induced by rTMS over rPPC.

**Methods:**

To do so, 14 healthy participants underwent resting state EEG recording before and after 30 min of rTMS at 1 Hz or sham stimulation over the rPPC (electrode position P6). Real stimulation was applied at 90% of motor threshold. Coherence values were computed on the electrodes nearby the stimulated site (i.e., P4, P8, and CP6) considering all possible inter- and intra-hemispheric combinations for the following frequency bands: delta (0.5–4 Hz), theta (4–8 Hz), alpha (8–12Hz), low beta (12–20 Hz), high beta (20–30 Hz), and gamma (30–50 Hz).

**Results and discussion:**

Results revealed a significant increase in coherence in delta, theta, alpha and beta frequency bands between rPPC and the contralateral homologous sites. Moreover, an increase in coherence in theta, alpha, beta and gamma frequency bands was found between rPPC and right frontal sites, reflecting the activation of the fronto-parietal network within the right hemisphere. Summarizing, subthreshold rTMS over rPPC revealed cortico-cortical inter- and intra-hemispheric connectivity as measured by the increase in coherence among these areas. Moreover, the present results further confirm previous evidence indicating that the increase of coherence values is related to intra- and inter-hemispheric inhibitory effects of rTMS. These results can have implications for devising evidence-based rehabilitation protocols after stroke.

## 1 Introduction

Repetitive transcranial magnetic stimulation (rTMS) is a non-invasive method to temporarily modulate neural activity (Silvanto and Muggleton, 2008; Miniussi et al., 2013) with long-lasting changes of cortical excitability (Robertson et al., 2003). Importantly, rTMS not only acts locally on interneural circuits underneath the coil but its effects also spread to functionally connected brain regions along cortico-cortical connections (Bortoletto et al., 2015; Siviero et al., 2023). When TMS is coupled with EEG, it is possible to map the network of cortical areas engaged in a specific cognitive function and give information on the functional coupling among brain areas with a very high temporal resolution (Ilmoniemi et al., 1997; Miniussi and Thut, 2010) thus providing information on the causal role of TMS-induced cortical changes on distant, but functionally interconnected, areas. The advantage of recording EEG before and after the delivery of a rTMS protocol is that of potentially providing information on the causal relationship between functionally connected areas in terms of both the temporal dynamics (pre- vs. post-stimulation) and the nature (excitatory vs. inhibitory) of the signal that is spread. That is, if an area A is active prior to area B, it can be assumed that area A and B are functionally connected, because a change in area A has an effect on area B, and that activity in area A causes a change in the activity of area B. Moreover, if area A, as an example, is inhibited by rTMS and a coherent reduction of activity is found in area B, the two areas are positively connected (i.e., excitatory connection), conversely if a reduction of activity in area A is followed by an enhancement of activity in area B, the two areas are negatively (i.e., inhibitory connection) connected (Miniussi and Thut, 2010).

Importantly, EEG can provide useful information in terms of brain oscillations which are a notable feature of brain activity and they are thought to play a central role in cognition (Wang, 2010; Tafuro et al., 2023). EEG signal can indeed be decomposed into frequency bands (Berger, 1929), each of which showing a specific power reflecting the amount of synchronized oscillatory activity expressed by neuronal ensembles oscillating in that specific frequency band. Importantly, the power of each frequency band can be used to calculate coherence, i.e., a measure reflecting the strength and the sign of the interplay between patterns of oscillating brain activity recorded at different locations on the scalp, and it is suggested to sustain effective communication among groups of neurons (Fries, 2005). Thus, high coherence between EEG signals recorded at different electrodes suggests high functional coupling between the underlying neuronal networks (i.e., functional connectivity). Coherence can be measured while participants are not performing any task (resting state) and it can, thus, reveal functional connectivity of areas in the resting state. Moreover, if resting state coherence is recorded prior and after the application of a rTMS protocol, it can give important insights on rTMS-induced perturbation effects on the state of the cortex in different areas of the brain, thus revealing both the network(s) of areas functionally connected with the stimulated site and the nature (excitatory vs. inhibitory) of this functional coupling. That is, if coherence among two sites is increased after inhibition of one of the two, it reflects an excitatory connection between the two sites (i.e., both sites are inhibited), conversely if coherence is decreased after the inhibition of one of the two, it reflects an inhibitory connection between the two sites (one site is inhibited and the other is excited).

From a translational perspective, the possibility to detect changes at the network level by means of measures of coherence in brain networks can pave the way for the development of effective interventions aimed at restoring cognitive deficits in stroke patients. Indeed, low-frequency rTMS of the right (contralesional) frontal cortex has been shown to reduce non-fluent aphasia symptoms (Shah et al., 2013), whereas low-frequency rTMS of the left (contralesional) parietal cortex has been shown to reduce symptoms (Hesse et al., 2011; Oliveri, 2011; Müri et al., 2013) in spatial neglect, i.e., a failure to report, respond, or orient to stimuli presented to the contralesional space (Heilman and Valenstein, 1979; Schenkenberg et al., 1980; Savazzi et al., 2007). The logic underlying the application of rTMS on the contralesional hemisphere stands on the assumptions of the interhemispheric rivalry model (Kinsbourne, 1977) stating that, in normal conditions, the two hemispheres inhibit each other. Accordingly, after a lesion to one hemisphere, the contralesional hemisphere is released from the inhibition exerted by the damaged one and becomes hyperactive. However, despite several pieces of evidence showing the efficacy of rTMS in restoring cognitive functions, little is known about the neural underpinnings allowing this to occur and different mechanisms are proposed for the amelioration of symptoms in aphasia and neglect patients. In this respect, amelioration of neglect symptoms, by applying rTMS to the contralesional hemisphere, has been invariably explained in terms of a reduction of the hyperactivity of the undamaged hemisphere and a restoration of the interhemispheric balance (Jacquin-Courtois, 2015). Conversely, amelioration of aphasia symptoms has been interpreted both as a restoration of the interhemispheric balance and as a reduction of maladaptive activation of areas within the right hemisphere (Gainotti, 2015). Importantly, most of the studies showing hyperactivity of the contralesional hemisphere in patients with neglect refers to sub-acute or chronic patients and, thus, leaving open the possibility, as proposed in aphasia literature, that the hyperactivity of the contralesional hemisphere might be the result of maladaptive plasticity (Umarova et al., 2011; Pia et al., 2012; Ricci et al., 2012; Bagattini et al., 2015b) as opposed to a release from inhibition caused by a lesion. Thus, understanding the role of the non-lesioned hemisphere in the genesis and maintenance of the deficits is important not only for a better comprehension of the neural plasticity and reorganization after stroke, but it will be crucial for devising more effective rehabilitation protocols (Bartolomeo, 2014; Lunven et al., 2015).

Several attempts to understand the interplay between the hemispheres to uncover the effects of hypo-activity (due to a lesion or caused by low frequency, inhibitory, rTMS) in one hemisphere on the functionality of the other (i.e., induced hyper-activation or hypo-activation) have been made (e.g., Koch et al., 2008, 2011, 2013; Salatino et al., 2014). However, none of them have used a direct approach by investigating coherence among brain areas with inhibitory TMS approaches for areas different from motor ones (e.g., Strens et al., 2002; Chen et al., 2003). To the best of our knowledge, such an approach to directly test the hypothesis that a reduction of activity in one hemisphere (right parietal lobe) would result in an enhancement of activity in homologous areas in the other hemisphere was present only in a paper by Bagattini et al. (2015b). In this paper, the authors applied 1 Hz rTMS in healthy participants to reduce cortical reactivity in one hemisphere and concurrently measured, with EEG, the effects of this reduction in functionally connected areas. They found that the sites contralateral to the stimulation exhibited a reduction in activity similar to that induced in the stimulated hemisphere. Similarly, adopting an interleaved TMS-fMRI approach, Ricci et al. (2012) showed that low frequency rTMS over right posterior parietal cortex (rPPC) caused decreased neural activity of parieto-frontal regions and of the homologous parietal regions of the left hemisphere. These results were, thus, in direct contrast to the hypothesis of a reciprocal inhibition of the two hemispheres suggesting that what is proposed for aphasia, i.e., maladaptive plasticity, could also account for spatial neglect.

Here, we extend previous investigations by means of an inhibitory rTMS protocol applied over rPPC and by recording resting state EEG before and after the application of rTMS. Coherence between the stimulated site and other cortical areas is used as a measure of functional connectivity to reveal both the functionally interconnected networks engaged by the stimulation and the nature of this connection. The aim of the present paper is, thus, that of revealing whether the reduction of cortical activity induced by 1 Hz rTMS in one hemisphere leads to a reduction (high coherence) of neural activity in other areas within the same hemisphere or on the contralateral hemisphere, thus providing evidence on cortico-cortical connectivity between rPPC and other brain areas. Implications for possible recruitment of intra- and inter-hemispheric neural pathways in spatial neglect are discussed.

## 2 Materials and methods

### 2.1 Participants

Twenty-one right-handed (as assessed with the Edinburgh Handedness Inventory; Oldfield, 1971) healthy volunteers (14 females), aged 19–33 years (mean 24.81 years, sd 3.39 years), took part in the experiment. They all had normal or corrected-to-normal visual acuity and no history of neurological or psychiatric disorders. All gave their written informed consent to participate in the experiment. The experiment was carried out according to the principles laid down in the 2013 Declaration of Helsinki and approved by the local Ethics Committee. The data from seven participants were not included in the analysis because of high noise in the EEG recordings or a too low number of segments for the power and coherence analysis (see below). Therefore, the research sample consisted of 14 participants (nine females; mean age 24.71 years, sd 3.41 years).

As assessed by a safety screening questionnaire (adapted from Rossi et al., 2011), the participants were negative for all the risk factors associated with TMS: none reported neurological disorders, cardiac pacemaker, any history of epilepsy or migraine, current treatment with any psychoactive medication or pregnancy.

### 2.2 Experimental design

**Figure 1:**
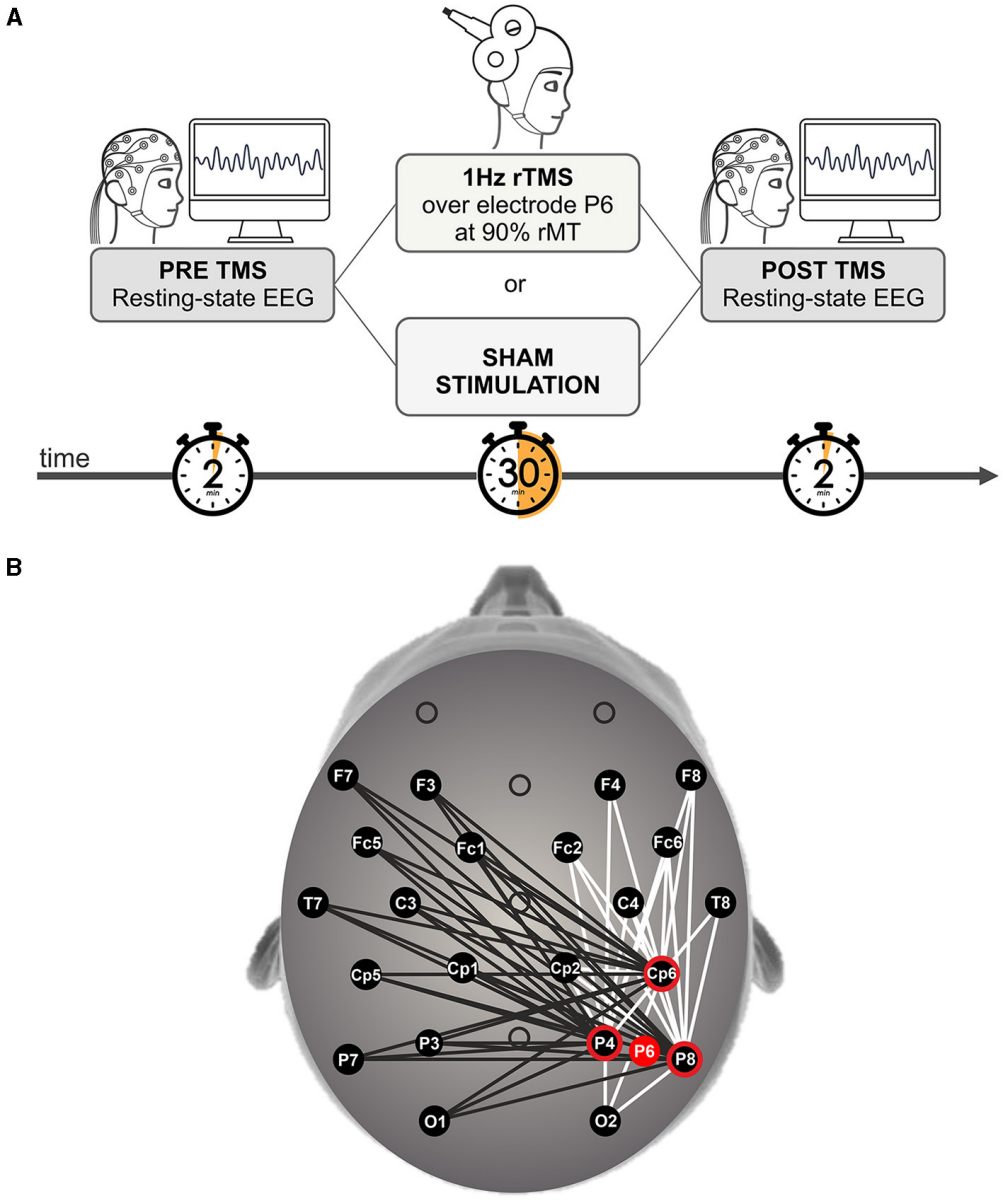
**(A)** Experimental design. Participants received either 30 min of 1 Hz repetitive transcranial magnetic stimulation (rTMS) or sham stimulation on right posterior parietal cortex (rPPC, electrode position P6), counterbalanced across participants. Notably, the stimulation was preceded and followed by 2 min of resting state EEG recording (i.e., PRE and POST TMS). During resting state sessions, participants were instructed to keep their eyes open while fixating a central cross displayed on the screen in front of them. **(B)** Topographical representation of all possible electrode pairs considered for calculating coherence values. The three nearest electrodes (P4, P8, and CP6, highlighted in red) to the stimulation site (i.e., P6, represented by a red spot) with all pairwise combinations with the remaining EEG channels (except for central ones—Fz, Cz and Pz- and Fp1 and Fp2) were taken into consideration. White lines refer to intra-hemispheric pairs of channels, while black lines refer to inter-hemispheric pairs of channels.

Figure 1A illustrates the experimental design. Two experimental sessions (TMS/Sham), the order of which was counterbalanced across participants, were conducted on two separate days. The sequence of each of the two experimental sessions was as the following. After placing the cap with electrodes for EEG recording, the motor threshold was assessed at the beginning of the first session to set the stimulation intensity (see TMS protocol section). Two minutes of resting-state EEG activity were then recorded, immediately before and after (hereafter called “PRE” and “POST”) the main stimulation protocols (TMS or Sham), which lasted for 30 min. Participants were tested in a dimly lit room, seated in a comfortable chair with their head stabilized by a chinrest and their eyes open while watching a fixation cross at the center of a black computer screen positioned at a viewing distance of about 57 cm.

In order to reduce uncontrolled effects of “mind wandering” or non-specific brain activity related to thoughts or mental imagery, which could affect the ongoing activity in the targeted systems and associated networks (Silvanto and Muggleton, 2008), the participants were instructed to relax and free their mind both during resting EEG recordings and stimulation. This procedure was adopted in both the stimulation protocols (TMS/Sham) to render all the protocols homogeneous with respect to the instructions.

### 2.3 TMS protocol

rTMS was delivered through a 70 mm figure-of-eight Magstim Air Film Coil connected with a Magstim Rapid^2^ system (maximum output 3.5 Tesla) (Magstim Company Limited, Whitland, UK). The stimulation was delivered at 1 Hz for 30 min (total number of pulses = 1,800) at 90% (mean 54.93% of Maximum Stimulator Output, MSO) of resting motor threshold (mean 61% of the MSO). These parameters have been shown to have an inhibitory effect on the stimulated cortex, that is to reduce cortical excitability for several minutes beyond the duration of the TMS trains (Maeda et al., 2000; Valero-Cabré et al., 2006; Bagattini et al., 2015b). Motor threshold was measured as the minimum stimulation intensity able to elicit a motor evoked potential (MEP) of ≥50 μV in the left first dorsal interosseous muscle in five of ten consecutive stimulations (Rossini et al., 1994). rTMS was applied unilaterally over the right posterior parietal cortex (rPPC) between P4 and P8 electrodes of the 10–20 International EEG system (i.e., at the position of the P6 electrode), corresponding to the right inferior parietal lobe (Fierro et al., 2000; Brighina et al., 2002; Bagattini et al., 2015b), that is the area mostly involved in the emergence of spatial neglect and, if stimulated with TMS, capable of inducing neglect-like symptoms in healthy individuals. The TMS coil was placed tangentially to the target scalp site with the handle pointing backwards, so as to induce a posterior-to-anterior current direction in the underlying cortical surface. To stabilize the coil in the targeted position and orientation with respect to the scalp, a mechanical arm (Magstim Articulated Coil Stand) was used, and the participants wore a custom-made collar for the entire duration of the stimulation protocol preventing any head movements. Moreover, the position of the coil was constantly checked by the experimenter and, on the rare occasions it was needed, corrected. The stimulation protocol (stimulus intensity, frequency and duration of the pulse train) was selected according to the international safety guidelines (Rossi et al., 2009) and commercial earplugs were used to protect the participants from the noise associated with TMS (Rossi et al., 2009). None of the participants reported negative effects during or after stimulation. For the Sham condition, we used the same parameters as for the rTMS session but, in order to reduce the intensity of the magnetic field reaching the scalp (Stokes et al., 2005), a custom-made 3-cm-thick block of polystyrene was placed between the coil and the scalp, thus, ensuring that the Sham stimulation was ineffective (Bagattini et al., 2015a; Mazzi et al., 2017).

### 2.4 EEG recording and preprocessing

TMS-compatible EEG equipment (BrainAmp, Brain Products GmbH, Munich, Germany) was used to record spontaneous EEG (BrainVision Recorder) in resting-state. EEG activity was continuously recorded from a Fast'n Easy cap with 27 TMS-compatible Ag/AgCl pellet pin electrodes (EasyCap GmbH, Herrsching, Germany) placed according to the 10-20 International System (O1, O2, P7, P3, Pz, P4, P8, CP5, CP1, CP2, CP6, T7, C3, Cz, C4, T8, FC5, FC1, FC2, FC6, F7, F3, Fz, F4, F8, Fp1, Fp2). Additional electrodes were used as reference, ground and for the electro-oculogram. The ground electrode was placed in AFz. All scalp channels were online referenced to the right mastoid (RM) and then re-referenced offline to the average of the RM and left mastoid (LM). Horizontal and vertical eye movements were recorded, respectively, with electrodes placed at the left and right canthi and above and below the right eye. The impedance of all the electrodes was kept below 5 KΩ. The EEG was recorded at 5,000 Hz sampling rate with a time constant of 10 s as low cut-off and a high cut-off of 1,000 Hz. The EEG signal was processed and analyzed off-line using Brain Vision Analyzer 2.1.

Continuous resting-state data were off-line down-sampled to 500 Hz. Brain components corresponding to ocular artifacts (such as eye blinks and eye movements) were identified and corrected using the ICA ocular correction (restricted Infomax) algorithm implemented in Brain Vision Analyzer 2 applied over to the whole continuous signal. Following ocular correction, data were filtered with a 0.5 Hz high-pass filter with a 24 dB/octave roll-off, as well as with a notch filter to remove power line noise (50 Hz). Segmentation into non-overlapping windows of 2.048 s was then applied to all channels (Kam et al., 2013) and epochs containing artifacts were rejected before further processing by means of a semiautomatic procedure. The length of the epoch was chosen to maintain consistency with previous literature (e.g., Wacker et al., 2009; Jamieson and Burgess, 2014; Ranlund et al., 2014; Ambrosini et al., 2020; Neuhaus et al., 2021; Grieder and Koenig, 2023) and, thus, allowing for easier comparison and integration of findings across studies. Moreover, the length of 2,048 ms allows for capturing multiple cycles of lower-frequency brain oscillations, provides a sufficient time window to analyze their dynamics accurately and it is an appropriate balance between temporal resolution and statistics. That is, it is short enough to capture rapid changes in brain activity, yet long enough to reduce noise and obtain more reliable estimates of brain activity features. Subsequently, a surface Laplacian transform was performed (reference-free current source density—CSD, Nunez et al., 1997) using spherical spline interpolation (Perrin et al., 1989). Only participants with at least 25 epochs per conditions, i.e., more than 50 s (Kam et al., 2013), were included in the subsequent analyses. Notably, no significant differences (all *p* > 0.05) in the average duration of artifact-free EEG data were found across conditions (pre TMS: M = 87 s, SD = 19.85, post TMS: M = 86 s, SD = 22.11, pre SHAM: M = 84 s, SD = 26.11, post SHAM: M = 83 s, SD = 19.56).

### 2.5 Power

EEG power spectrum was estimated for all frequency bins between 0.5 and 50 Hz using *Fast Fourier Transform* (FFT, resolution 0.5 Hz, *Hanning window 10%*) and then averaged across epochs under the same conditions for all electrodes. Mean absolute power was then calculated for the following frequency bands: delta (0.5–4 Hz), theta (4–8 Hz), alpha (8–12 Hz), low beta (12–20 Hz), high beta (20–30 Hz) and gamma (30–50 Hz).

Regional power changes were determined using the Stimulation-Related Power (SRPow) for each condition (TMS/Sham) and each electrode position according to the following Equation (1):


(1) $$\begin{array}{l} \text{SRPo}{\text{w}}_{\text{x}}\left(\%\right)=\left[\left(\text{POST Po}{\text{w}}_{\text{x}}-\text{PRE Po}{\text{w}}_{\text{x}}\right)/\text{PRE Po}{\text{w}}_{\text{x}}\right]\times 100 \end{array}$$

Therefore, the so calculated SRPow of an electrode “x” represents the percentage change of spectral power after the stimulation (i.e., POST) when compared to a baseline (i.e., PRE, data collected before the stimulation) at a certain frequency band. As a consequence, increases in power from the baseline (i.e., PRE) to the POST stimulation phase correspond to a positive value, whereas negative values reflect decreases in power. This event-related desynchronization/synchronization procedure (Pfurtscheller and Lopes da Silva, 1999) allowed to reduce the effects of inter-subject variability in absolute spectral power values (Gerloff et al., 1998; Hummel et al., 2002).

### 2.6 Coherence

EEG absolute power spectra were then used to calculate coherence. Coherence between two EEG signals (*x* and *y*) at each frequency *f* was then computed with BrainVision Analyzer 2 according to the following Equation (2), which represents an extension of the Pearson's correlation coefficient to complex number pairs:


(2) $${\text{Coh}}_{\text{xy}}(f){\text{=|CS}}_{\text{xy}}(f){\text{|}}^{2}\text{/}[{\text{|CS}}_{\text{xx}}(f){\text{||CS}}_{\text{yy}}(f)\text{|}]$$

Here CS_xy_ (*f* ) corresponds to the cross-spectrum, while CS_xx_ (*f* ) and CS_yy_ (*f* ) are the autospectrum estimates of the x and y signals, respectively. Accordingly, the coherence value results in a number ranging between 0 and 1, with 0 indicating the lack of functional connectivity between two signals at a given frequency band, and 1 meaning a strong interregional interaction. Coherence values (see Supplementary material) were then pooled into the same frequency band defined as follows: delta (0.5–4 Hz), theta (4–8 Hz), alpha (8–12 Hz), low beta (12–20 Hz), high beta (20–30 Hz), and gamma (30–50 Hz).

In order to test for changes in connectivity induced by rTMS, the calculation of coherence was performed by considering the three nearest electrodes to the stimulation site (i.e., P4, P8, CP6) with all possible intra- and inter-hemispheric combinations (Figure 1B). Specifically, intra-hemispheric coherence (namely, within the stimulated hemisphere) was assessed on the right hemisphere considering the electrodes of interest in combination with O2, CP2, T8, C4, FC6, FC2, F8, and F4. Similarly, inter-hemispheric coherence (namely, reflecting functional connectivity between the stimulated site and the contralateral hemisphere) was assessed considering the same three electrodes of interest in combination with O1, P7, P3, CP5, CP1, T7, C3, FC5, FC1, F7, and F3 (all placed on the left hemisphere).

Stimulation-Related Coherence (SRCoh, Plewnia et al., 2008) was then calculated for each stimulation condition (TMS and Sham) by means of the Equation (3):


(3) $${\text{SRCoh}}_{\text{xy}}\text{= POST }{\text{Coh}}_{\text{xy}}-\text{PRE }{\text{Coh}}_{\text{xy}}$$

Therefore, positive values indicate a stronger coherence as a consequence of the stimulation protocol. Conversely, SRCoh has a negative value when coherence is lower after the stimulation than before. The present procedure has, thus, the dual advantage of (1) normalizing for the effect of the baseline coherence level and (2) reducing the effect of inter-subject and inter-electrode variability of absolute spectral coherence introduced by the reference electrodes (Fein et al., 1988; Rappelsberger and Petsche, 1988).

### 2.7 Statistical analysis

Statistical comparisons between conditions were performed on SRPow and SRCoh values using a series of one-tailed paired *t*-tests with bootstrapping in order to reveal reliable increments of these measures after rTMS compared to Sham.

Specifically, as far as concerns power, rTMS SRPow values for each electrode were compared to Sham SRPow values in all investigated frequency bands (δ, θ, α, low β, high β, γ). Taking into account coherence, rTMS SRCoh values were compared to Sham SRCoh values in all the considered frequency bands. The difference in SRPow and SRCoh values for the two stimulation conditions (rTMS/Sham) was analyzed by means of a non-parametric Monte Carlo percentile bootstrap simulation (Efron and Tibshirani, 1993; Oruç et al., 2011) implemented into a custom-made Matlab (MathWorks, Natick, MA) script. This procedure creates a simulated data distribution by re-sampling the data with replacement. To this end, 50,000 re-samples for the rTMS minus Sham conditions of both SRPow and SRCoh values were used. The lower 5th percentile of the re-sampled data distribution served as the critical values for the one-tailed 0.05 significance level. If the 5th percentile results to be above the zero level (rTMS > Sham), it means that values of SRPow and SRCoh are significantly larger for the rTMS condition than the Sham condition. This analysis was performed separately for SRPow and SRCoh values, electrode/electrode pairs (see Figure 1B) and frequency bands (δ, θ, α, low β, high β, γ).

## 3 Results

### 3.1 Power

**Figure 2:**
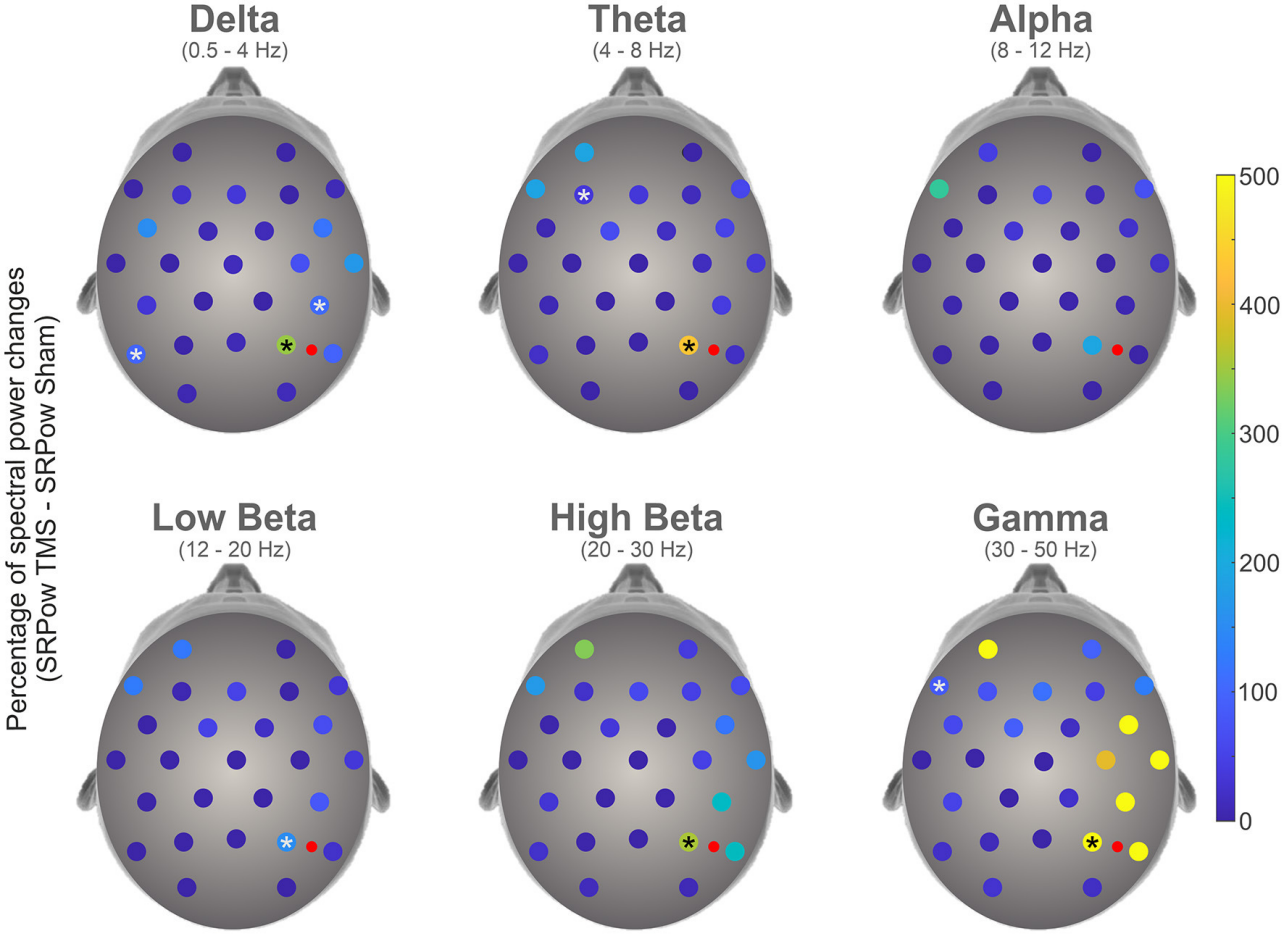
Topographical representation of percentage changes of spectral power following 1 Hz rTMS compared to Sham condition in the different frequency bands. Asterisks represent the electrodes where a significant enhancement has been highlighted by a bootstrap analysis. Red dots represent the stimulation site.

Figure 2 represents the increment of SRPow after rTMS compared to Sham, for each electrode as a function of the frequency bands. Bootstrap analysis revealed a significant enhancement of power (in percentage of change) following rTMS compared to Sham condition in electrodes P4 (345.88, *p* < 0.0001), CP6 (106.95, *p* < 0.0001) and P7 (98.76, *p* < 0.0001) in Delta band, P4 (430.81, *p* < 0.0001) and F3 (36.79, *p* < 0.0001) in Theta, P4 in both Low (153.90, *p* < 0.0001) and High (355.96, *p* < 0.0001) Beta and P4 (499.07, 0.0001) and F7 (84.56, *p* < 0.0001) in Gamma domain. Statistical analysis did not yield any significant differences in the alpha band.

### 3.2 Coherence

Statistical results for intra- and inter-hemispheric coherence are, respectively, reported in Tables 1 and 2 as assessed by bootstrap tests on SRCoh data (rTMS SRCoh vs. Sham SRCoh) for each electrode pair in the six different frequency bands considered. As expected from the literature (e.g., Capotosto et al., 2014), intra-hemispheric coherence was in mean higher (0.068) than the inter-hemispheric coherence (0.037). This was evident both in general and for the single frequency bands where coherence values were found significant in both electrode-pair arrangements: theta (intra: 0.042; inter: 0.036), alpha (intra: 0.062; inter: 0.043), low beta (intra: 0.064; inter: 0.020), and high beta (intra: 0.100; inter: 0.035).

**Table 1:** Results of the bootstrap analysis comparing rTMS SRCoh vs. Sham SRCoh condition concerning intra-hemispheric coherence data.

**Intra-hemispheric coherence**	**O2**	**CP2**	**T8**	**C4**	**FC6**	**FC2**	**F8**	**F4**
**Delta (0.5–4 Hz)**								
P4	0.043	0.003	0.011	0.002	0.003	−0.017	0.014	0.027
	0.18	0.48	0.28	0.48	0.45	0.80	0.27	0.07
P8	0.010	0.026	0.021	0.008	−0.039	0.004	−0.005	0.005
	0.35	0.19	0.17	0.41	0.85	0.45	0.55	0.47
CP6	−0.012	0.035	0.023	0.058	−0.005	0.025	0.001	0.031
	0.58	0.22	0.18	0.14	0.60	0.17	0.47	0.10
**Theta (4–8 Hz)**								
P4	0.035	0.029	**0.028**	0.019	**0.051**	0.011	**0.060**	0.020
	0.14	0.10	**0.04**	0.13	**0.02**	0.20	**0.01**	0.16
P8	**0.030**	0.009	**0.052**	0.009	−0.008	−0.010	0.016	0.002
	**0.04**	0.34	**0.0001**	0.30	0.71	0.78	0.23	0.44
CP6	−0.012	0.019	0.011	0.038	0.005	0.002	**0.034**	−0.002
	0.74	0.17	0.34	0.13	0.36	0.45	**0.046**	0.59
**Alpha (8–12 Hz)**								
P4	**0.072**	0.039	**0.044**	0.026	**0.066**	−0.016	**0.073**	0.011
	**0.03**	0.08	**0.04**	0.16	**0.01**	0.67	**0.01**	0.33
P8	0.030	0.018	**0.055**	0.022	0.022	0.009	−0.003	0.014
	0.24	0.35	**0.04**	0.29	0.28	0.38	0.56	0.24
CP6	0.030	0.028	0.049	0.021	0.029	0.004	**0.056**	−0.021
	0.17	0.21	0.10	0.32	0.19	0.45	**0.02**	0.79
**Low beta (12–20 Hz)**								
P4	**0.029**	−0.014	0.014	0.009	**0.038**	−0.011	**0.069**	0.018
	**0.03**	0.75	0.31	0.32	**0.01**	0.61	**0.001**	0.19
P8	0.045	0.022	0.108	0.061	0.053	0.025	−0.011	0.040
	0.14	0.25	0.07	0.14	0.14	0.11	0.60	0.11
CP6	0.043	0.035	**0.104**	0.060	0.061	−0.004	**0.077**	0.038
	0.13	0.10	**0.01**	0.15	0.10	0.59	**0.004**	0.14
**High beta (20–30 Hz)**								
P4	0.050	0.019	0.022	0.054	0.047	0.012	0.073	0.012
	0.11	0.26	0.37	0.12	0.11	0.31	0.10	0.36
P8	0.072	0.049	0.122	0.087	0.054	0.028	0.049	0.038
	0.12	0.14	0.05	0.05	0.20	0.13	0.23	0.19
CP6	0.068	0.058	**0.107**	0.071	**0.087**	0.025	0.071	0.026
	0.09	0.07	**0.03**	0.09	**0.04**	0.16	0.07	0.22
**Gamma (30–50 Hz)**								
P4	0.039	0.059	0.023	0.067	0.022	0.065	0.094	0.013
	0.28	0.10	0.39	0.11	0.34	0.05	0.06	0.40
P8	0.071	0.070	0.070	**0.091**	0.066	0.048	0.030	0.027
	0.16	0.10	0.20	**0.048**	0.11	0.12	0.33	0.26
CP6	0.083	**0.132**	0.095	0.085	**0.102**	**0.098**	0.072	0.026
	0.11	**0.01**	0.096	0.11	**0.03**	**0.02**	0.06	0.28

The value above in each cell corresponds to the difference between rTMS SRCoh and Sham SRCoh while the value below refers to the exact *p*-value obtained. Significant results are highlighted in red bold font.

**Table 2:** Results of the bootstrap analysis comparing rTMS SRCoh vs. Sham SRCoh condition concerning inter-hemispheric coherence data.

**Inter-hemispheric coherence**	**O1**	**P7**	**P3**	**CP5**	**CP1**	**T7**	**C3**	**FC5**	**FC1**	**F7**	**F3**
**Delta (0.5–4 Hz)**											
P4	−0.010	0.000	0.049	−0.014	0.051	−0.048	0.006	−0.043	0.010	0.018	0.007
	0.55	0.48	0.12	0.66	0.10	0.97	0.48	0.89	0.36	0.38	0.41
P8	0.018	0.007	0.016	0.010	0.038	−0.019	0.008	0.002	−0.033	−0.005	−0.01
	0.23	0.43	0.27	0.37	0.07	0.77	0.40	0.48	0.84	0.55	0.61
CP6	−0.029	0.031	0.025	0.046	**0.067**	−0.043	0.031	−0.027	−0.017	0.002	−0.006
	0.67	0.24	0.31	0.15	**0.01**	0.79	0.26	0.65	0.84	0.48	0.50
**Theta (4–8 Hz)**											
P4	0.020	**0.027**	0.042	0.017	**0.046**	0.000	0.030	−0.029	0.013	0.017	0.001
	0.27	**0.01**	0.06	0.17	**0.0004**	0.51	0.18	0.77	0.21	0.33	0.46
P8	−0.003	0.020	0.017	0.015	−0.013	−0.008	0.026	0.007	−0.017	0.027	0.000
	0.57	0.17	0.09	0.26	0.84	0.64	0.11	0.39	0.84	0.07	0.54
CP6	−0.014	0.021	**0.051**	0.020	**0.031**	−0.025	0.020	−0.024	**0.024**	0.017	−0.028
	0.61	0.14	**0.03**	0.17	**0.046**	0.69	0.23	0.67	**0.01**	0.31	0.66
**Alpha (8–12 Hz)**											
P4	0.019	0.012	0.047	0.051	**0.053**	0.009	0.040	0.012	0.019	0.030	0.017
	0.25	0.32	0.08	0.09	**0.04**	0.37	0.13	0.32	0.34	0.21	0.29
P8	0.014	−0.003	**0.042**	−0.008	0.010	−0.028	0.008	0.007	0.034	0.041	0.009
	0.37	0.53	**0.04**	0.62	0.38	0.87	0.40	0.39	0.10	0.12	0.32
CP6	−0.006	0.016	**0.044**	**0.044**	0.021	−0.031	0.048	−0.014	0.018	0.046	−0.007
	0.60	0.26	**0.045**	**0.03**	0.23	0.89	0.10	0.59	0.27	0.18	0.55
**Low beta (12–20Hz)**											
**P4**	−0.005	0.001	0.023	0.016	0.024	−0.024	0.033	−0.029	−0.005	0.014	−0.015
	0.56	0.45	0.05	0.17	0.06	0.97	0.20	0.88	0.67	0.34	0.66
**P8**	−0.007	0.012	0.005	0.001	0.000	0.007	−0.006	−0.007	0.012	−0.006	−0.017
	0.63	0.07	0.33	0.48	0.51	0.30	0.67	0.67	0.15	0.63	0.89
**CP6**	−0.008	0.015	**0.022**	**0.019**	**0.022**	−0.025	0.017	−0.038	0.009	0.005	−0.045
	0.57	0.26	**0.02**	**0.03**	**0.05**	0.88	0.19	0.85	0.22	0.43	0.90
**High beta (20–30 Hz)**											
P4	−0.011	−0.002	0.024	−0.008	0.034	−0.020	0.012	−0.040	−0.007	0.038	−0.029
	0.62	0.52	0.08	0.61	0.11	0.77	0.39	0.85	0.61	0.23	0.74
P8	0.029	0.021	−0.003	0.014	0.019	−0.002	0.008	0.004	−0.018	0.022	−0.023
	0.26	0.15	0.55	0.16	0.12	0.54	0.21	0.36	0.76	0.24	0.94
CP6	−0.002	0.032	0.012	**0.033**	0.030	−0.012	**0.043**	−0.021	−0.008	0.028	−0.034
	0.53	0.09	0.30	**0.04**	0.07	0.65	**0.03**	0.64	0.64	0.25	0.72
**Gamma (30–50 Hz)**											
P4	−0.005	0.015	0.036	0.021	0.051	0.002	0.028	−0.034	0.017	0.050	0.002
	0.52	0.33	0.19	0.32	0.08	0.48	0.30	0.74	0.25	0.22	0.48
P8	0.021	0.012	0.001	0.001	0.008	−0.011	−0.012	−0.010	−0.015	0.010	−0.023
	0.34	0.38	0.54	0.45	0.31	0.63	0.90	0.69	0.70	0.34	0.85
CP6	0.015	0.036	0.048	0.044	0.035	0.003	0.036	−0.028	0.002	0.042	−0.033
	0.39	0.11	0.14	0.08	0.11	0.46	0.22	0.68	0.45	0.24	0.72

The value above in each cell corresponds to the difference between rTMS SRCoh and Sham SRCoh while the value below refers to the exact *p*-value obtained. Significant results are highlighted in red bold font.

**Figure 3:**
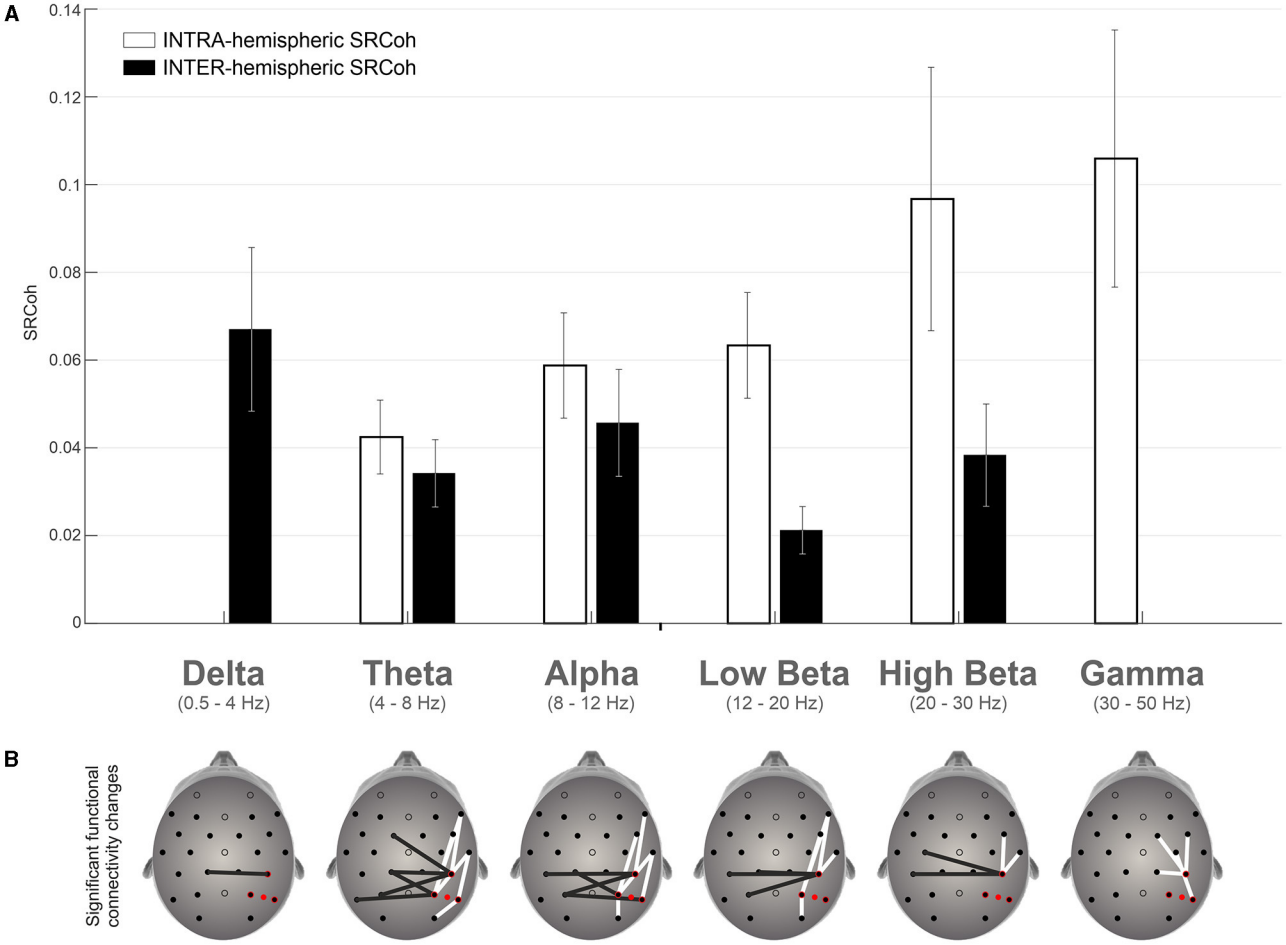
**(A)** Mean differences between TMS SRCoh and Sham SRCoh for significant electrode pairs across the frequency bands considered for intra- and inter-hemispheric coherence data respectively. Vertical lines represent the SEM. **(B)** Topographical representation of the electrode pairs with a significant increase of coherence. White bars refer to intra-hemispheric pairs of channels, while black bars refer to inter-hemispheric pairs of channels.

Figure 3 summarizes the significant effects in terms of coherence change due to active vs. sham stimulation of the right hemisphere and provides a clear topographical representation of such effects. Interestingly, intra- and inter-hemispheric coherence increase behaved in a different manner with respect to the frequency bands: the former decreased while the latter increased as the frequency band increased (Figure 3A).

## 4 Discussion

The present study investigated the effects of repetitive transcranial magnetic stimulation (rTMS) on functional connectivity through coherence in the brain using EEG. The results contribute to our understanding of the neural mechanisms underlying the therapeutic effects of rTMS and its potential application in stroke rehabilitation. Specifically, we investigated intra- and inter-hemispheric coherence changes induced by rTMS of rPPC. Healthy participants were administered with a 30 min of low frequency (1Hz) rTMS protocol, which has been shown to reduce neural excitability (Chen et al., 1997; Boroojerdi et al., 2000), while resting-state EEG signal was recorded before and after rTMS. The main goal of the present investigation was to uncover the networks of areas functionally interconnected with the stimulated (rPPC) area by measuring EEG coherence. The logic was the following: higher coherence between rPPC and other distant areas would imply that these areas are functionally connected with rPPC and that their activity after rTMS is changed in the same manner (functional coupling) as the activity in rPPC. Specifically, we found that the sites showing a functional coupling (increased coherence) with rPPC after rTMS are the homologous (parietal) sites on the contralateral (left) hemisphere, in line with previous findings using TMS-evoked potentials (Bagattini et al., 2015b), and fronto-central sites within the stimulated (right) hemisphere. Accordingly, and increase in power was found at the stimulated sites and at the contralateral homologous and frontal sites. Since the effects of this rTMS protocol applied to rPPC have been assessed to be inhibitory by directly measuring cortical reactivity (Bagattini et al., 2015b), we can infer that, after rTMS, sites showing high coherence with rPPC are inhibited as well, i.e., they have a reduced cortical reactivity.

These results have implications both for basic knowledge about how different areas are functionally connected and for the possibility of applying this knowledge to the field of rehabilitation after brain damage. Firstly, they reveal a network of functionally coupled areas, both within and between hemispheres, oscillating coherently. Specifically, the present results revealed a functionally coupled fronto-parietal network within the right hemisphere, a network that has been found to be relevant for several cognitive functions (Marek and Dosenbach, 2018), such as for example attentional performance (Fellrath et al., 2016; Rogala et al., 2020), visuo-motor processes (Naranjo et al., 2006; Iturrate et al., 2018), visuospatial judgement (Guidali et al., 2023), and working memory and cognitive control (Gulbinaite et al., 2014; Bertaccini et al., 2022). Moreover, results have shown a functional coupling among homologous sites in the two hemispheres which are, thus, found to be connected in an excitatory manner, in line with previous research with different neuroimaging techniques in both healthy participants and neglect patients (Ricci et al., 2012; Bagattini et al., 2015b; Lunven et al., 2015; Killington et al., 2016; Ptak et al., 2020; Schintu et al., 2020, 2021). Importantly, cortico-cortical connectivity changes induced by TMS on resting-state connectivity MRI (Fox et al., 2012) have also been investigated. These studies have shown to be a powerful tool to reveal complex patterns of network modulation both within and across hemispheres. Unfortunately though, very few TMS protocols have been applied to areas other than the motor cortex (Han et al., 2023). Since network changes induced by TMS are specific for the stimulated area (Castrillon et al., 2020), a direct comparison with the present data lacks conclusive power. To our knowledge, the study most similar to the one presented here relates to a recently published TMS-EEG and MRI integrated approach (Esposito et al., 2022). In this study, the authors applied TMS, among other areas, to the right parietal cortex and analyzed structural and functional connectivity of the default mode network. The results have shown strong functional coupling of the right parietal sites with the homologous left parietal sites and with the fronto-central sites, in agreement with the present data. More research is needed to obtain a clearer picture of the complexity of cortico-cortical connectivity changes induced by TMS.

Interestingly, results also show higher inter-hemispheric coherence for low-frequency bands and higher intra-hemispheric coherence for high-frequency bands which can be explained by the underlying neural mechanisms and functional connectivity patterns in the brain (Varela et al., 2001; Buzsáki and Draguhn, 2004; Fries, 2015). Indeed, frequency power spectrum is topographically organized as a gradient along the antero-posterior axis: lower frequency bands dominate at back of the brain while higher frequency bands dominate at frontal sites (Niedermeyer, 1999). It is, thus, more likely that, by applying rTMS over the rPPC, more inter-hemispheric coherence can be found between posterior pairs of electrodes at low frequency bands. In the same vein, an increase of intra-hemispheric coherence for higher frequency bands is expected along the fronto-parietal network within the stimulated (right) hemisphere. Moreover, low-frequency bands, such as delta and theta waves, are associated with slower oscillations and are believed to reflect long-range connectivity and coordination between brain regions. These slow waves facilitate communication and synchronization between distant brain areas, including inter-hemispheric connections. Therefore, higher inter-hemispheric coherence in low-frequency bands suggests stronger coordination and information exchange between the two hemispheres. Consistently, a recent study showed that theta band effective inter-hemispheric connectivity between parietal regions sustained the leftward visuospatial advantage typically observed in neurologically healthy individuals (Bagattini et al., 2022). On the other hand, high-frequency bands, such as beta and gamma waves, are associated with faster oscillations and are believed to reflect local processing and information integration within specific brain regions. These fast waves are important for intra-hemispheric communication and are involved in various cognitive functions. Therefore, higher intra-hemispheric coherence in high-frequency bands indicates enhanced local processing and functional integration within each hemisphere.

Importantly, the present results provide implications for stroke rehabilitation, particularly addressing cognitive deficits such as neglect. Indeed, previous research has demonstrated the efficacy of contralesional low-frequency rTMS in reducing symptoms associated with these conditions. However, the neural mechanisms underlying these improvements have not been fully understood. The present study, by revealing the network of functionally connected areas engaged by rTMS and the nature of this connection, provided insights into the interplay between the hemispheres and the potential mechanisms underlying the genesis of the chronicity of symptoms. Specifically, the notion of the hyperactivity of the left hemisphere as a consequence of the release from the inhibition exerted by the right hemisphere (Kinsbourne, 1977) may not be the primary cause of the genesis of neglect symptoms. Rehabilitation protocols based on this notion, thus, may not be effective in reducing neglect symptoms in the long term (Müri et al., 2013; Carter and Barrett, 2023). Conversely, these data and those already present in literature on neglect patients could serve in devising *ah-hoc* protocols taking into account the complex interplay of intra- and inter-hemispheric networks abnormalities in neglect patients (Bartolomeo, 2014, 2019, 2021; Bartolomeo and Thiebaut de Schotten, 2016). Indeed, the hyperactivity in the left hemisphere of neglect patients could be the result of maladaptive plasticity (Nava and Röder, 2011; Altman et al., 2019) following brain lesion and not as the cause of the breakdown of the reciprocal inhibition exerts by the two hemispheres, which seems to be an oversimplification of brain dynamics (Berlucchi, 1983; Ptak et al., 2020). Accordingly, contralesional hyperactivity may not be adequate as the only determinant to implement rehabilitation protocols. Indeed, imaging studies have found no signs of hyperactivity in the left hemisphere of neglect patients tested in the acute phase (Vallar et al., 1988; Fiorelli et al., 1991; Perani et al., 1993; Umarova et al., 2011, 2014) whereas right hemisphere fronto-parietal network activity has been shown to predict neglect's severity (Corbetta and Shulman, 2002; He et al., 2007; Carter et al., 2010; Machner et al., 2020). These pieces of evidence, thus, support the conclusion that the reinstatement of neural functionality in both the left and right hemispheres contributes as a predictor of functional recovery from neglect (Corbetta et al., 2005; Cramer, 2008; Lunven et al., 2015; Umarova et al., 2016).

In conclusion, the study demonstrated that inhibitory rTMS applied to the right posterior parietal cortex modulated functional connectivity and coherence in the brain. The findings challenge the notion of reciprocal inhibition between the hemispheres and suggest a role for maladaptive plasticity in spatial neglect. The study's approach of investigating non-motor areas provides valuable insights into the intra- and inter-hemispheric effects of rTMS and its potential as a therapeutic intervention in stroke rehabilitation. Further research with larger sample sizes and stroke patients is warranted to confirm and expand upon these findings.

## Data availability statement

The datasets presented in this study can be found in online repositories. The names of the repository/repositories and accession number(s) can be found below: https://doi.org/10.17605/OSF.IO/4VHM7.

## Ethics statement

The studies involving humans were approved by Ethical Committee of the Department of Neuroscience, Biomedicine and Movement Sciences. The studies were conducted in accordance with the local legislation and institutional requirements. The participants provided their written informed consent to participate in this study.

## Author contributions

CM: Data curation, Formal analysis, Methodology, Software, Supervision, Validation, Visualization, Writing – review & editing, Investigation, Writing – original draft. SM: Data curation, Methodology, Supervision, Validation, Visualization, Writing – review & editing, Investigation, Software. CB: Supervision, Validation, Visualization, Writing – review & editing, Data curation, Investigation, Methodology, Software. JS-L: Supervision, Validation, Visualization, Writing – review & editing, Data curation, Investigation, Methodology, Software. SS: Methodology, Software, Supervision, Validation, Visualization, Writing – review & editing, Conceptualization, Funding acquisition, Project administration, Writing – original draft.
