# Supplementary material for: Coherent activity within and between hemispheres: cortico-cortical connectivity revealed by rTMS of the right posterior parietal cortex
Source: Front Hum Neurosci. 2024 Mar 7;18:1362742. doi: 10.3389/fnhum.2024.1362742 (PMC10954802; doi:10.3389/fnhum.2024.1362742)
Supplement: Supplementary file 1 [file Data_Sheet_1.PDF]

Table 1: pre TMS INTRA coherence

INTRA-HEMISPHERIC COHERENCE	O2	CP2	T8	C4	FC6	FC2	F8	F4
**Delta (0.5-4Hz)**								
**P4**	0.152	0.093	0.057	0.068	0.070	0.052	0.047	0.044
**P8**	0.081	0.061	0.085	0.072	0.088	0.050	0.071	0.054
**CP6**	0.087	0.065	0.076	0.093	0.053	0.045	0.063	0.042
**Theta (4-8Hz)**								
**P4**	0.093	0.061	0.045	0.044	0.044	0.033	0.037	0.034
**P8**	0.038	0.044	0.045	0.047	0.066	0.037	0.049	0.035
**CP6**	0.055	0.043	0.075	0.079	0.037	0.041	0.041	0.041
**Alpha (8-12Hz)**								
**P4**	0.135	0.127	0.067	0.065	0.085	0.067	0.058	0.059
**P8**	0.061	0.086	0.106	0.085	0.092	0.083	0.099	0.064
**CP6**	0.060	0.061	0.076	0.134	0.069	0.084	0.062	0.081
**Low Beta (12-20Hz)**								
**P4**	0.087	0.077	0.073	0.058	0.044	0.047	0.037	0.046
**P8**	0.068	0.055	0.115	0.056	0.067	0.049	0.078	0.051
**CP6**	0.055	0.041	0.066	0.142	0.058	0.070	0.046	0.047
**High Beta (20-30Hz)**								
**P4**	0.118	0.059	0.142	0.068	0.074	0.044	0.091	0.070
**P8**	0.124	0.063	0.175	0.071	0.103	0.047	0.132	0.078
**CP6**	0.096	0.043	0.093	0.143	0.078	0.063	0.088	0.065
**Gamma (30-50Hz)**								
**P4**	0.133	0.0560	0.222	0.0790	0.0890	0.0550	0.108	0.0870
**P8**	0.158	0.0720	0.232	0.0900	0.0970	0.0520	0.142	0.0940
**CP6**	0.124	0.0620	0.109	0.158	0.0950	0.0540	0.0880	0.0750

Table 2: pre TMS INTER coherence

INTER-HEMISPHERIC COHERENCE	O1	P7	P3	CP5	CP1	T7	C3	FC5	FC1	F7	F3
**Delta (0.5-4Hz)**											
**P4**	0.098	0.089	0.085	0.090	0.074	0.092	0.087	0.079	0.058	0.082	0.080
**P8**	0.058	0.106	0.065	0.074	0.060	0.072	0.072	0.066	0.073	0.078	0.073
**CP6**	0.094	0.077	0.087	0.072	0.051	0.099	0.102	0.084	0.053	0.094	0.088
**Theta (4-8Hz)**											
**P4**	0.059	0.043	0.063	0.061	0.040	0.060	0.059	0.078	0.041	0.058	0.074
**P8**	0.063	0.059	0.047	0.049	0.045	0.053	0.047	0.052	0.051	0.047	0.051
**CP6**	0.074	0.050	0.058	0.059	0.040	0.079	0.066	0.079	0.033	0.068	0.096
**Alpha (8-12Hz)**											
**P4**	0.064	0.079	0.082	0.076	0.088	0.071	0.072	0.066	0.068	0.065	0.059
**P8**	0.075	0.095	0.062	0.081	0.075	0.079	0.082	0.067	0.061	0.059	0.053
**CP6**	0.064	0.083	0.068	0.062	0.070	0.073	0.072	0.096	0.061	0.089	0.094
**Low Beta (12-20Hz)**											
**P4**	0.052	0.047	0.047	0.035	0.044	0.049	0.039	0.062	0.041	0.059	0.062
**P8**	0.056	0.046	0.044	0.034	0.044	0.039	0.032	0.044	0.039	0.052	0.045
**CP6**	0.064	0.048	0.037	0.033	0.042	0.056	0.044	0.075	0.039	0.071	0.079
**High Beta (20-30Hz)**											
**P4**	0.066	0.048	0.041	0.043	0.036	0.057	0.043	0.067	0.041	0.069	0.068
**P8**	0.054	0.051	0.047	0.044	0.036	0.051	0.039	0.052	0.044	0.058	0.055
**CP6**	0.074	0.049	0.038	0.038	0.045	0.055	0.048	0.080	0.036	0.078	0.090
**Gamma (30-50Hz)**											
**P4**	0.084	0.051	0.054	0.049	0.049	0.053	0.059	0.079	0.046	0.079	0.082
**P8**	0.055	0.048	0.046	0.042	0.038	0.047	0.047	0.052	0.040	0.057	0.051
**CP6**	0.079	0.039	0.040	0.038	0.053	0.044	0.056	0.080	0.041	0.081	0.096

Table 3: post TMS INTRA coherence

INTRA-HEMISPHERIC COHERENCE	O2	CP2	T8	C4	FC6	FC2	F8	F4
**Delta (0.5-4Hz)**								
**P4**	0.137	0.096	0.057	0.082	0.049	0.049	0.058	0.059
**P8**	0.076	0.080	0.101	0.087	0.087	0.057	0.101	0.068
**CP6**	0.076	0.092	0.079	0.14	0.056	0.065	0.089	0.070
**Theta (4-8Hz)**								
**P4**	0.090	0.084	0.056	0.056	0.064	0.055	0.072	0.058
**P8**	0.050	0.060	0.079	0.047	0.067	0.039	0.080	0.045
**CP6**	0.042	0.068	0.080	0.105	0.051	0.049	0.086	0.058
**Alpha (8-12Hz)**								
**P4**	0.162	0.129	0.103	0.105	0.113	0.077	0.119	0.083
**P8**	0.092	0.10	0.164	0.124	0.152	0.088	0.131	0.074
**CP6**	0.090	0.113	0.122	0.178	0.102	0.102	0.125	0.094
**Low Beta (12-20Hz)**								
**P4**	0.087	0.071	0.076	0.064	0.058	0.048	0.079	0.061
**P8**	0.090	0.076	0.185	0.104	0.118	0.065	0.090	0.077
**CP6**	0.087	0.076	0.138	0.184	0.107	0.070	0.102	0.078
**High Beta (20-30Hz)**								
**P4**	0.111	0.077	0.118	0.088	0.091	0.052	0.112	0.066
**P8**	0.125	0.099	0.222	0.119	0.129	0.060	0.139	0.080
**CP6**	0.11	0.086	0.156	0.179	0.128	0.072	0.132	0.067
**Gamma (30-50Hz)**								
**P4**	0.122	0.095	0.20	0.106	0.111	0.078	0.157	0.090
**P8**	0.155	0.126	0.263	0.15	0.151	0.076	0.168	0.104
**CP6**	0.129	0.136	0.18	0.211	0.15	0.090	0.159	0.087

Table 4: post TMS INTER coherence

INTER-HEMISPHERIC COHERENCE	O1	P7	P3	CP5	CP1	T7	C3	FC5	FC1	F7	F3
**Delta (0.5-4Hz)**											
**P4**	0.063	0.076	0.082	0.056	0.117	0.046	0.091	0.036	0.055	0.097	0.079
**P8**	0.070	0.107	0.057	0.061	0.093	0.059	0.076	0.066	0.049	0.075	0.061
**CP6**	0.058	0.098	0.077	0.081	0.116	0.050	0.107	0.052	0.047	0.105	0.075
**Theta (4-8Hz)**											
**P4**	0.055	0.057	0.071	0.059	0.077	0.050	0.068	0.048	0.058	0.081	0.066
**P8**	0.056	0.066	0.049	0.046	0.042	0.046	0.047	0.049	0.045	0.079	0.035
**CP6**	0.043	0.065	0.079	0.072	0.072	0.051	0.064	0.043	0.062	0.091	0.063
**Alpha (8-12Hz)**											
**P4**	0.091	0.074	0.101	0.119	0.098	0.074	0.113	0.069	0.106	0.101	0.086
**P8**	0.109	0.095	0.097	0.091	0.096	0.068	0.091	0.081	0.101	0.122	0.062
**CP6**	0.080	0.078	0.087	0.101	0.108	0.064	0.112	0.089	0.096	0.137	0.082
**Low Beta (12-20Hz)**											
**P4**	0.045	0.048	0.053	0.049	0.063	0.036	0.069	0.038	0.050	0.074	0.058
**P8**	0.066	0.063	0.050	0.039	0.043	0.041	0.041	0.041	0.049	0.051	0.036
**CP6**	0.057	0.063	0.053	0.046	0.063	0.038	0.064	0.042	0.049	0.078	0.043
**High Beta (20-30Hz)**											
**P4**	0.063	0.052	0.053	0.042	0.067	0.044	0.062	0.040	0.044	0.101	0.050
**P8**	0.072	0.056	0.049	0.037	0.056	0.055	0.039	0.045	0.039	0.079	0.032
**CP6**	0.072	0.056	0.054	0.036	0.072	0.047	0.065	0.041	0.045	0.089	0.041
**Gamma (30-50Hz)**											
**P4**	0.071	0.065	0.071	0.049	0.083	0.051	0.069	0.044	0.049	0.111	0.070
**P8**	0.072	0.072	0.055	0.041	0.051	0.060	0.038	0.043	0.040	0.075	0.038
**CP6**	0.079	0.073	0.064	0.048	0.078	0.052	0.072	0.045	0.049	0.108	0.050

Table 5: pre SHAM INTRA coherence

INTRA-HEMISPHERIC COHERENCE	O2	CP2	T8	C4	FC6	FC2	F8	F4
**Delta (0.5-4Hz)**								
**P4**	0.136	0.0700	0.0520	0.0390	0.0600	0.0370	0.0400	0.0370
**P8**	0.0520	0.0400	0.0690	0.0490	0.0570	0.0330	0.0620	0.0340
**CP6**	0.0370	0.0490	0.0940	0.0910	0.0380	0.0480	0.0460	0.0350
**Theta (4-8Hz)**								
**P4**	0.108	0.0870	0.0590	0.0480	0.0730	0.0390	0.0660	0.0370
**P8**	0.0710	0.0460	0.0630	0.0520	0.0530	0.0350	0.0500	0.0410
**CP6**	0.0480	0.0470	0.0850	0.100	0.0430	0.0530	0.0420	0.0320
**Alpha (8-12Hz)**								
**P4**	0.163	0.162	0.0880	0.0880	0.113	0.0670	0.0770	0.0690
**P8**	0.0860	0.0780	0.107	0.0780	0.0830	0.0790	0.0800	0.0750
**CP6**	0.0800	0.0720	0.113	0.155	0.0750	0.0900	0.0620	0.0900
**Low Beta (12-20Hz)**								
**P4**	0.116	0.0810	0.0850	0.0710	0.0810	0.0550	0.0870	0.0550
**P8**	0.0890	0.0570	0.199	0.0900	0.0960	0.0610	0.101	0.0710
**CP6**	0.0670	0.0550	0.119	0.174	0.0840	0.0630	0.0920	0.0710
**High Beta (20-30Hz)**								
**P4**	0.141	0.0810	0.146	0.0960	0.104	0.0630	0.139	0.0630
**P8**	0.165	0.0770	0.287	0.134	0.145	0.0640	0.169	0.0970
**CP6**	0.123	0.0720	0.160	0.207	0.122	0.0790	0.117	0.0750
**Gamma (30-50Hz)**								
**P4**	0.164	0.106	0.263	0.154	0.119	0.105	0.211	0.0880
**P8**	0.212	0.101	0.329	0.191	0.170	0.0860	0.184	0.130
**CP6**	0.187	0.136	0.209	0.268	0.172	0.131	0.138	0.0940

Table 6: pre SHAM INTER coherence

INTER-HEMISPHERIC COHERENCE	O1	P7	P3	CP5	CP1	T7	C3	FC5	FC1	F7	F3
**Delta (0.5-4Hz)**											
**P4**	0.0680	0.0460	0.102	0.0530	0.0450	0.0380	0.0530	0.0380	0.0450	0.0370	0.0430
**P8**	0.0460	0.0690	0.0560	0.0560	0.0340	0.0420	0.0480	0.0590	0.0390	0.0490	0.0470
**CP6**	0.0430	0.0490	0.0730	0.0820	0.0350	0.0420	0.0610	0.0510	0.0350	0.0490	0.0470
**Theta (4-8Hz)**											
**P4**	0.0630	0.0450	0.0760	0.0500	0.0470	0.0520	0.0540	0.0390	0.0300	0.0370	0.0430
**P8**	0.0570	0.0520	0.0520	0.0570	0.0400	0.0370	0.0590	0.0530	0.0360	0.0340	0.0510
**CP6**	0.0490	0.0490	0.0690	0.0610	0.0450	0.0410	0.0580	0.0510	0.0400	0.0370	0.0420
**Alpha (8-12Hz)**											
**P4**	0.0710	0.0810	0.113	0.0820	0.119	0.0720	0.0730	0.0920	0.0700	0.0650	0.0660
**P8**	0.0870	0.0830	0.0810	0.0740	0.0690	0.0710	0.0680	0.0840	0.0690	0.0700	0.0660
**CP6**	0.0640	0.0910	0.0970	0.0870	0.0630	0.0510	0.0800	0.0780	0.0640	0.0720	0.0700
**Low Beta (12-20Hz)**											
**P4**	0.0410	0.0440	0.0580	0.0420	0.0480	0.0350	0.0460	0.0400	0.0430	0.0380	0.0380
**P8**	0.0520	0.0550	0.0380	0.0470	0.0500	0.0480	0.0410	0.0490	0.0520	0.0440	0.0430
**CP6**	0.0380	0.0480	0.0430	0.0530	0.0500	0.0370	0.0470	0.0430	0.0450	0.0380	0.0400
**High Beta (20-30Hz)**											
**P4**	0.0490	0.0450	0.0690	0.0460	0.0560	0.0440	0.0490	0.0410	0.0450	0.0460	0.0420
**P8**	0.0740	0.0800	0.0400	0.0830	0.0560	0.0500	0.0680	0.0800	0.0470	0.0560	0.0660
**CP6**	0.0580	0.0780	0.0490	0.0880	0.0600	0.0480	0.0810	0.0810	0.0410	0.0610	0.0690
**Gamma (30-50Hz)**											
**P4**	0.0680	0.0570	0.0850	0.0770	0.0680	0.0570	0.0760	0.0540	0.0720	0.0690	0.0690
**P8**	0.0770	0.0590	0.0470	0.0600	0.0540	0.0470	0.0550	0.0630	0.0510	0.0560	0.0560
**CP6**	0.0780	0.0600	0.0800	0.0890	0.0620	0.0580	0.0780	0.0630	0.0560	0.0700	0.0680

Table 7: post SHAM INTRA coherence

INTRA-HEMISPHERIC COHERENCE	O2	CP2	T8	C4	FC6	FC2	F8	F4
**Delta (0.5-4Hz)**								
**P4**	0.0780	0.0690	0.0400	0.0500	0.0360	0.0510	0.0370	0.0250
**P8**	0.0370	0.0330	0.0650	0.0550	0.0950	0.0360	0.0980	0.0430
**CP6**	0.0390	0.0420	0.0740	0.0800	0.0450	0.0430	0.0710	0.0310
**Theta (4-8Hz)**								
**P4**	0.0700	0.0810	0.0420	0.0410	0.0420	0.0500	0.0410	0.0410
**P8**	0.0520	0.0520	0.0460	0.0430	0.0620	0.0480	0.0660	0.0480
**CP6**	0.0470	0.0530	0.0780	0.0880	0.0520	0.0590	0.0530	0.0520
**Alpha (8-12Hz)**								
**P4**	0.119	0.126	0.0800	0.101	0.0750	0.0920	0.0660	0.0810
**P8**	0.0870	0.0740	0.110	0.0950	0.121	0.0750	0.115	0.0710
**CP6**	0.0800	0.0950	0.110	0.178	0.0790	0.104	0.0690	0.124
**Low Beta (12-20Hz)**								
**P4**	0.0880	0.0890	0.0730	0.0680	0.0560	0.0670	0.0600	0.0520
**P8**	0.0660	0.0560	0.160	0.0770	0.0950	0.0520	0.124	0.0570
**CP6**	0.0570	0.0550	0.0860	0.156	0.0710	0.0670	0.0720	0.0630
**High Beta (20-30Hz)**								
**P4**	0.0850	0.0790	0.100	0.0620	0.0730	0.0590	0.0860	0.0470
**P8**	0.0940	0.0640	0.213	0.0950	0.116	0.0500	0.126	0.0610
**CP6**	0.0690	0.0570	0.117	0.172	0.0840	0.0630	0.0900	0.0510
**Gamma (30-50Hz)**								
**P4**	0.114	0.0860	0.219	0.114	0.120	0.0620	0.166	0.0780
**P8**	0.138	0.0860	0.290	0.159	0.158	0.0620	0.181	0.113
**CP6**	0.109	0.0780	0.186	0.237	0.126	0.0690	0.136	0.0790

Table 8: post SHAM INTER coherence

INTER-HEMISPHERIC COHERENCE	O1	P7	P3	CP5	CP1	T7	C3	FC5	FC1	F7	F3
**Delta (0.5-4Hz)**											
**P4**	0.0420	0.0330	0.0490	0.0340	0.0370	0.0400	0.0520	0.0380	0.0320	0.0330	0.0360
**P8**	0.0410	0.0620	0.0310	0.0320	0.0280	0.0490	0.0430	0.0560	0.0480	0.0510	0.0450
**CP6**	0.0360	0.0390	0.0370	0.0450	0.0330	0.0350	0.0350	0.0470	0.0450	0.0580	0.0400
**Theta (4-8Hz)**											
**P4**	0.0380	0.0320	0.0420	0.0320	0.0380	0.0420	0.0340	0.0390	0.0340	0.0430	0.0340
**P8**	0.0530	0.0380	0.0370	0.0390	0.0500	0.0370	0.0340	0.0440	0.0470	0.0390	0.0360
**CP6**	0.0320	0.0430	0.0400	0.0530	0.0450	0.0380	0.0350	0.0390	0.0450	0.0420	0.0370
**Alpha (8-12Hz)**											
**P4**	0.0790	0.0630	0.0840	0.0740	0.0760	0.0660	0.0740	0.0820	0.0890	0.0710	0.0760
**P8**	0.106	0.0860	0.0740	0.0930	0.0800	0.0870	0.0680	0.0900	0.0740	0.0910	0.0670
**CP6**	0.0870	0.0710	0.0720	0.0820	0.0790	0.0720	0.0730	0.0850	0.0800	0.0740	0.0650
**Low Beta (12-20Hz)**											
**P4**	0.0390	0.0440	0.0400	0.0410	0.0440	0.0460	0.0420	0.0450	0.0570	0.0380	0.0490
**P8**	0.0690	0.0590	0.0390	0.0510	0.0500	0.0430	0.0570	0.0530	0.0500	0.0490	0.0510
**CP6**	0.0400	0.0480	0.0370	0.0460	0.0490	0.0430	0.0500	0.0480	0.0460	0.0400	0.0480
**High Beta (20-30Hz)**											
**P4**	0.0560	0.0520	0.0570	0.0530	0.0520	0.0510	0.0560	0.0540	0.0540	0.0400	0.0540
**P8**	0.0640	0.0640	0.0450	0.0620	0.0570	0.0560	0.0600	0.0680	0.0600	0.0560	0.0660
**CP6**	0.0580	0.0530	0.0530	0.0520	0.0580	0.0520	0.0540	0.0640	0.0570	0.0430	0.0540
**Gamma (30-50Hz)**											
**P4**	0.0600	0.0570	0.0660	0.0560	0.0510	0.0530	0.0580	0.0530	0.0580	0.0520	0.0560
**P8**	0.0720	0.0710	0.0570	0.0580	0.0600	0.0710	0.0580	0.0640	0.0660	0.0630	0.0660
**CP6**	0.0640	0.0570	0.0550	0.0560	0.0520	0.0620	0.0590	0.0550	0.0620	0.0550	0.0560
